# MicroRNA-181a suppresses parkin-mediated mitophagy and sensitizes neuroblastoma cells to mitochondrial uncoupler-induced apoptosis

**DOI:** 10.18632/oncotarget.9786

**Published:** 2016-06-02

**Authors:** Min Cheng, Lei Liu, Yuanzhi Lao, Weijie Liao, Meijian Liao, Xuan Luo, Jiangbin Wu, Weidong Xie, Yaou Zhang, Naihan Xu

**Affiliations:** ^1^ School of Life Sciences, Tsinghua University, Beijing 100084, China; ^2^ Key Lab in Healthy Science and Technology, Division of Life Science, Graduate School at Shenzhen, Tsinghua University, Shenzhen 518055, China; ^3^ School of Pharmacy, Shanghai University of Traditional Chinese Medicine, Shanghai 201203, China; ^4^ Department of Chemistry, Tsinghua University, Beijing 100084, P.R. China; ^5^ Open FIESTA Center, Tsinghua University, Shenzhen 518055, P.R. China

**Keywords:** microRNA, mitochondria, mitophagy, parkin, apoptosis

## Abstract

Damage to mitochondria often results in the activation of both mitophagy and mitochondrial apoptosis. The elimination of dysfunctional mitochondria is necessary for mitochondrial quality maintenance and efficient energy supply. Here we report that miR-181a is a novel inhibitor of mitophagy. miR-181a is downregulated by mitochondrial uncouplers in human neuroblastoma SH-SY5Y cells. Overexpression of miR-181a inhibits mitochondrial uncoupling agents-induced mitophagy by inhibiting the degradation of mitochondrial proteins without affecting global autophagy. Knock down of endogenous miR-181a accelerates the autophagic degradation of damaged mitochondria. miR-181a directly targets Parkin E3 ubiquitin ligase and partially blocks the colocalization of mitochondria and autophagosomes/lysosomes. Re-expression of exogenous Parkin restores the inhibitory effect of miR-181a on mitophagy. Furthermore, miR-181a increases the sensitivity of neuroblastoma cells to mitochondrial uncoupler-induced apoptosis, whereas miR-181a antagomir prevents cell death. Because mitophagy defects are associated with a variety of human disorders, these findings indicate an important link between microRNA and Parkin-mediated mitophagy and highlights a potential therapeutic strategy for human diseases.

## INTRODUCTION

Mitochondria are multifunctional cellular organelles, which are primarily responsible for energy production *via* oxidative phosphorylation in the inner mitochondrial membrane. Mitochondria have major roles in cell survival, cell death and cell signaling pathways [1]. The mitochondrial network is a dynamic and adaptable system that must remain healthy in order to meet the various needs of host cells in response to the bioenergetic and environmental changes [2, 3] Disturbances in mitochondrial homoeostasis result in damaged and dysfunctional mitochondrial network, leading to a range of human diseases, including neurodegeneration and cancer [4–6]. The elimination of dysfunctional mitochondria is indispensible for mitochondrial quality maintenance and efficient energy supply survival. Damaged mitochondria are targeted for degradation *via* two major catabolic processes, the ubiquitin-proteasome system for eliminating mitochondrial outer membrane proteins and the autophagy-lysosome pathway for degrading mitochondria as whole organelles [7, 8]. The latter process also selectively excludes damaged mitochondria *via* a specific autophagic pathway called mitophagy [9].

Recent studies have identified specific regulators of mitophagy including PINK1/Parkin and mitophagy receptors in mammalian systems [10–14]. The genes encoding PINK1 (PTEN-induced putative kinase 1) and Parkin are found to be mutated in certain forms of autosomal recessive Parkinson's disease (PD) [15–18]. Genetic studies in *Drosophila* suggest that both PINK1 and Parkin are required for mitochondrial integrity, loss of either protein results in mitochondrial dysfunction, leading to the degeneration of flight muscles and dopaminergic neurons [19, 20]. PINK1 is a mitochondrial serine/threonine kinase that functions as a mitochondrial stress sensor. In healthy mitochondria, PINK1 is imported into the mitochondrial inner membrane where it is rapidly degraded by the inner membrane presenilin-associated rhomboid-like protease PARL and the mitochondrial-processing protease (MPP) [21–23]. Depolarization of the mitochondria with either chemical or protein uncouplers leads to the accumulation of PINK1 which is stabilized on the outer mitochondrial surface, where it induces the translocation of Parkin from the cytosol to damaged mitochondria *via* PINK1-mediated phosphorylation of Parkin [23, 24]. Following its recruitment to the mitochondria, Parkin promotes the degradation of diverse mitochondrial membrane and matrix proteins *via* its E3 ubiquitin ligase activity [12, 25]. Increasing evidence has suggested a major role for mitophagy receptors in the clearance of mitochondria. The elimination of mitochondria in reticulocytes is mediated by a BCL-2 related mitochondrial outer membrane protein NIX (also known as BNIP3L). NIX contains a conserved LC3-binding motif LIR (LC3-interacting region) and may act as a receptor for targeting mitochondria to autophagosomes. The function of NIX is not restricted to erythrocyte maturation, as depolarization of the mitochondria also enhances NIX and LC3 interactions in HeLa cells [14, 26, 27]. A recent study identified the mitochondrial outer membrane protein FUNDC1 as a mitochondrial receptor for hypoxia-induced mitophagy [13]. FUNDC1 binds to LC3 through a conserved LIR motif and this interaction is enhanced under hypoxic conditions.

Studies of the past several years have demonstrated that microRNAs regulate a wide range of biological processes, including autophagy. Many miRNAs have since been well characterized in the modulation of different autophagic stages [28, 29]. However, the roles of miRNAs in regulating mitophagy are not well understood. It has been reported that deletion of Kap1, a cofactor of KRAB-containing zinc finger proteins (KRAB-ZFPs), in erythroblasts fails to induce mitophagy-associated genes and retained mitochondria because of the persistent expression of miRNAs targeting mitophagy transcripts [30]. A recent study reported that miR-137, a hypoxia-responsive miRNA, inhibits hypoxia-induced mitophagy by targeting two mitophagy receptors, FUNDC1 and NIX [31].

It has been reported that miR-181a inhibits starvation and rapamycin-induced autophagy *via* targeting ATG5 and sensitized gastric cancer cells to cisplatin [32, 33]. miR-181a also promotes anoikis by suppressing autophagy in a mammary epithelial cell line MCF10A. The effects of miR-181a on anoikis can be reversed by overexpression of ATG5 [34]. In this study we report that miR-181a is a novel mitophagy inhibitor. miR-181a is significantly downregulated by mitochondrial uncouplers FCCP and CCCP. miR-181a suppresses mitophagy by targeting Parkin E3 ubiquitin ligase. The inhibitory effect of miR-181a on mitophagy can be rescued by the re-expression of exogenous Parkin. miR-181a also sensitizes neuroblastoma cells to mitochondrial uncoupler-induced apoptosis.

## RESULTS

### Mitochondrial uncouplers downregulate miR-181a expression

**Figure 1:**
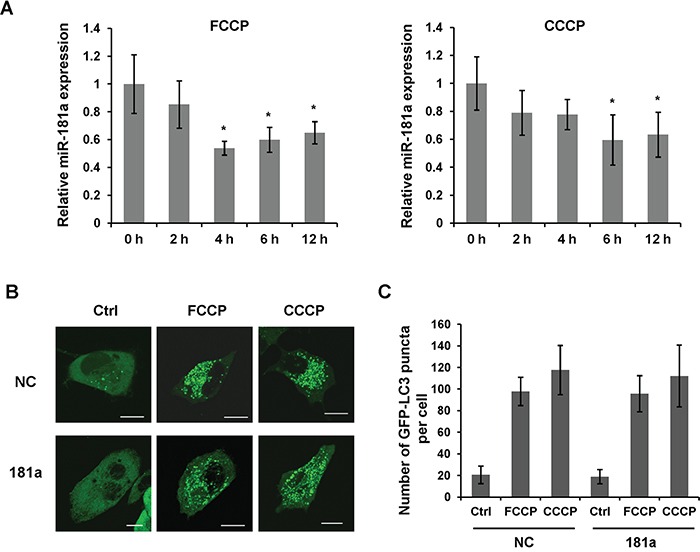
miR-181a is downregulated by mitochondrial uncouplers **A.** Analysis of miR-181a expression in human neuroblastoma SH-SY5Y cells. Cells were treated with 10 μM FCCP or CCCP for indicated time pointes. Data shown are from five independent experiments, *p<0.05. **B.** GFP-LC3 puncta formation assay in SH-SY5Y cells overexpressing miR-181a or negative control miRNA (NC). Cells were treated with 10 μM FCCP or CCCP for 6 h. The distribution of GFP-LC3 was captured by confocal microscope. Scale bar, 10 μm. **C.** Quantification the number of GFP-LC3 puncta in the cells (n = 100 cells from three independent experiments).

Carbonylcyanide-4-trifluorometh-oxyphenylhydrazone (FCCP) and carbonylcyanide-3-chlorophenylhydra-zone (CCCP) are mitochondrial uncouplers that are commonly used to induce mitophagy and the PINK1/Parkin pathway [35]. Both chemicals are nonspecific ionophores that cause a severe loss of mitochondrial membrane potential followed by the recruitment of LC3 to the mitochondria. To investigate the role of miR-181a in mitochondrial uncoupler-induced mitophagy, we firstly detected the expression of miR-181a in a human neuroblastoma cell line SH-SY5Y subjected to FCCP and CCCP treatment. The level of miR-181a started to decrease at 2 h after the initiation of drug treatment, was significantly reduced at 6 h and maintained at a relatively low level until 12 h after treatment (Figure 1A). We also examined the expression of miR-181a in human glioblastoma cell line A172 and primary human neurons (HN). Both FCCP and CCCP induced a significant reduction of miR-181a in these cell lines (Supplementary Figure S1). Using LC3 as a marker of autophagy activation, we found a dramatic increase of GFP-LC3 puncta after FCCP or CCCP treatment in control cells, whereas miR-181a did not affect global LC3 puncta accumulation upon drug treatment (Figure 1B & 1C).

### MiR-181a suppresses mitochondrial uncoupler-induced mitophagy

**Figure 2:**
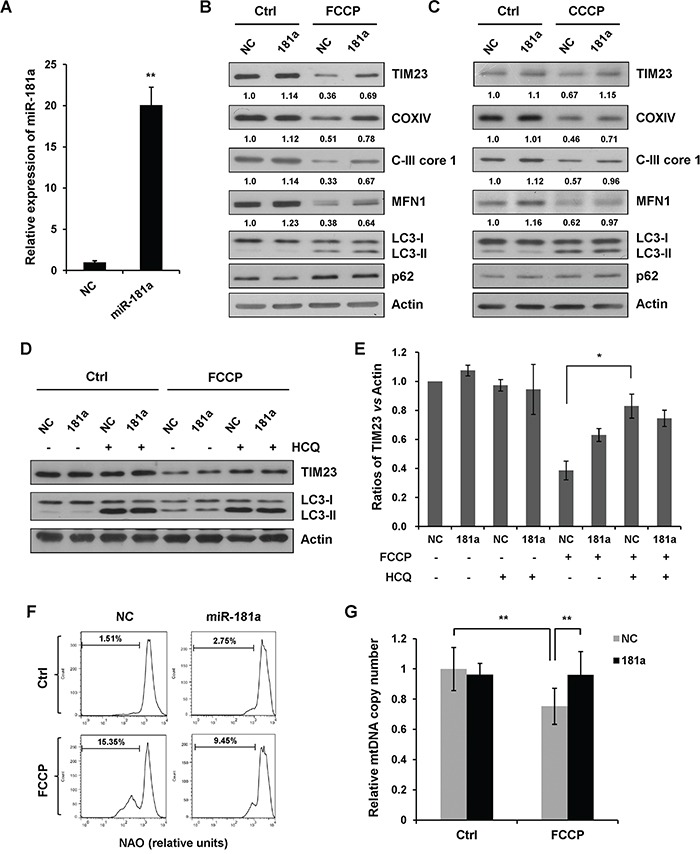
miR-181a inhibits mitochondrial uncoupler-induced mitophagy **A.** The relative expression of miR-181a in SH-SY5Y cells transfected with miR-181a mimics or the negative control (NC). **B.** SH-SY5Y cells transfected with miR-181a or NC were treated with 10 μM FCCP or **C.** CCCP for 12 h. Samples were collected for western blot to analyze the expression of TIM23, COXIV, C-III core, MFN1, LC3, p62, and Actin. Densitometric ratios of mitochondrial marker proteins are quantified by Image J software. **D.** SH-SY5Y cells transfected with NC or miR-181a were exposed to FCCP (10 μM) and HCQ (20 μM) for 12 h. Western blot was performed to analyze the status of TIM23, LC3 and Actin. **E.** Image J densitometric analysis of the TIM23/Actin ratios from immunoblots is shown (data from three independent experiments, *p<0.05). **F.** Mitochondrial content after 12 h of treatment with 10 uM FCCP was assessed by flow cytometry for NAO. **G.** Quantification of mitochondrial DNA copy number in NC or miR-181a transfected cells treated with FCCP (data from three independent experiments, **p<0.01).

Mitophagy can be monitored biochemically by measuring the degradation of mitochondrial proteins from the inner membrane, outer membrane and mitochondrial matrix. Here, we used mitochondrial proteins TIM23, COXIV, and respiratory chain complex III (C-III) subunit core 1 as a readout for mitophagy. Immunoblotting revealed that overexpression of miR-181a suppressed mitochondrial uncoupler-induced degradation of TIM23, COXIV, and C-III core 1 without affecting the LC3-I to LC3-II transition (Figure 2A–2C). Previous studies have shown that ubiquitination of mitofusins MFN1 is induced by Parkin E3 ligase upon membrane depolarization and leads to the degradation in a proteasome-dependent manner upstream of mitophagy [36–38]. Both FCCP and CCCP induced a marked reduction of MFN1 in control miRNA (NC) transfected cells, whereas miR-181a inhibited MFN1 degradation upon drug treatment. Although p62 is widely used as a biochemical marker for general autophagy, it is not a reliable marker for mitochondrial uncoupler-induced mitophagy in SH-SY5Y cells (Figure 2B & 2C). We also determined the effect of miR-181a on mitophagy in A172 cells. Similarly, overexpression of miR-181a attenuated FCCP-induced mitochondrial protein degradation (Supplementary Figure S2A).

To demonstrate that mitochondria were degraded by an autophagic process, we treated cells with a lysosomotropic inhibitor hydroxychloroquine (HCQ) to prevent endosomal acidification and autophagosome-lysosome fusion. HCQ significantly reversed FCCP-induced loss of TIM23 in control cells without markedly affecting TIM23 levels in miR-181a transfected cells. This suggested that miR-181a inhibits FCCP-induced mitochondrial degradation by autophagy (Figure 2D & 2E). Next, we assessed mitochondrial content by flow cytometry for Nonyl acridine orange (NAO) that fluorescences independently of mitochondrial membrane potential. FCCP-induced mitochondrial clearance was markedly lower in miR-181a transfected cells than in the control cells (Figure 2F). To verify these results, we also analyzed the number of mitochondrial DNA copies by real-time PCR. FCCP-induced loss of mtDNA was significantly prevented by miR-181a (Figure 2G).

### MiR-181a partially inhibits colocalization of mitochondria with autophagosomes/lysosomes

**Figure 3:**
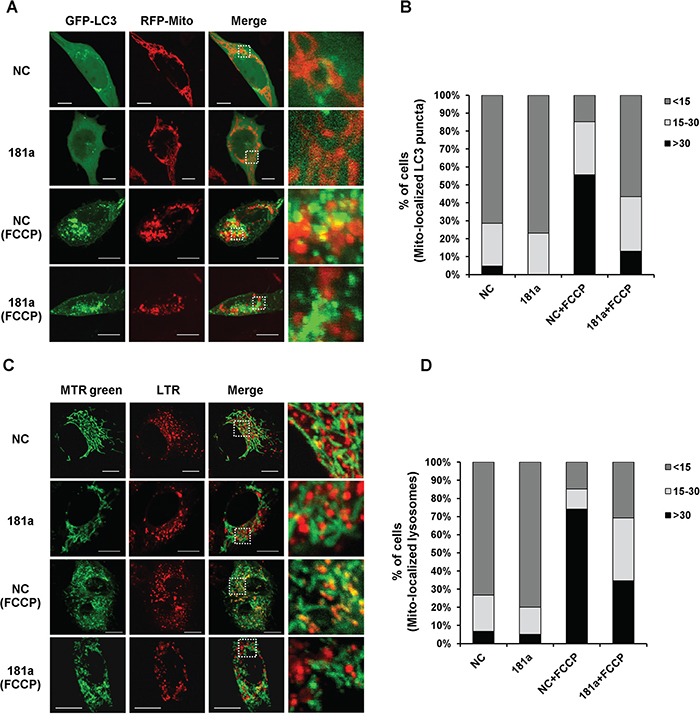
miR-181a partially blocks colocalization of mitochondria with autophagosomes/lysosomes **A.** SH-SY5Y cells overexpressing miR-181a or NC were co-transfected with GFP-LC3 and Mito-RFP for 24 h, followed by incubation with 10 μM FCCP for 6 h. The distribution of GFP-LC3 and Mito-FRP was visualized by confocal microscopy. Scale bar, 10 μm. **B.** Quantitative analysis of cells that contain fragmented mitochondria-localized LC3 puncta (values represent mean number of puncta counts per cell, n=100 cells from three independent experiments). **C.** Representative confocal images of MitoTracker Green FM (MTR green) and LysoTracker Red (LTR) in SH-SY5Y cells treated with or without FCCP for 6 h. Scale bar, 10 μm. **D.** Quantitative analysis of cells that contain fragmented mitochondria-localized lysosomes (n=100 from three independent experiments).

To assess the potential colocalization between mitochondria and autophagosomes after mitochondrial depolarization, SH-SY5Y cells were co-transfected with GFP-LC3 and mito-RFP. Colocalization between mitochondria and of LC3-labelled autophagosomes was present in NC transfected cells after FCCP treatment, whereas miR-181a markedly decreased the colocazation (Figure 3A). Quantification of LC3 puncta that colocalized with mitochondria demonstrated a significant difference in the percentage of cells with more than 30 mitochondrial-localized LC3 puncta between control and miR-181a transfected cells after mitochondrial depolarization (Figure 3B). Damaged mitochondria are incorporated into autophagosomes and delivered to lysosomes for degradation; therefore, we examined colocalization between mitochondria and lysosomes after mitochondrial depolarization. Mitochondria and lysosomes were labeled with MitoTracker Green FM and LysoTracker Red respectively. Similarly, miR-181a decreased the colocalization of mitochondria with lysosomes in response to FCCP treatment (Figure 3C & 3D).

### Blockage of endogenous miR-181a accelerates mitophagy

**Figure 4:**
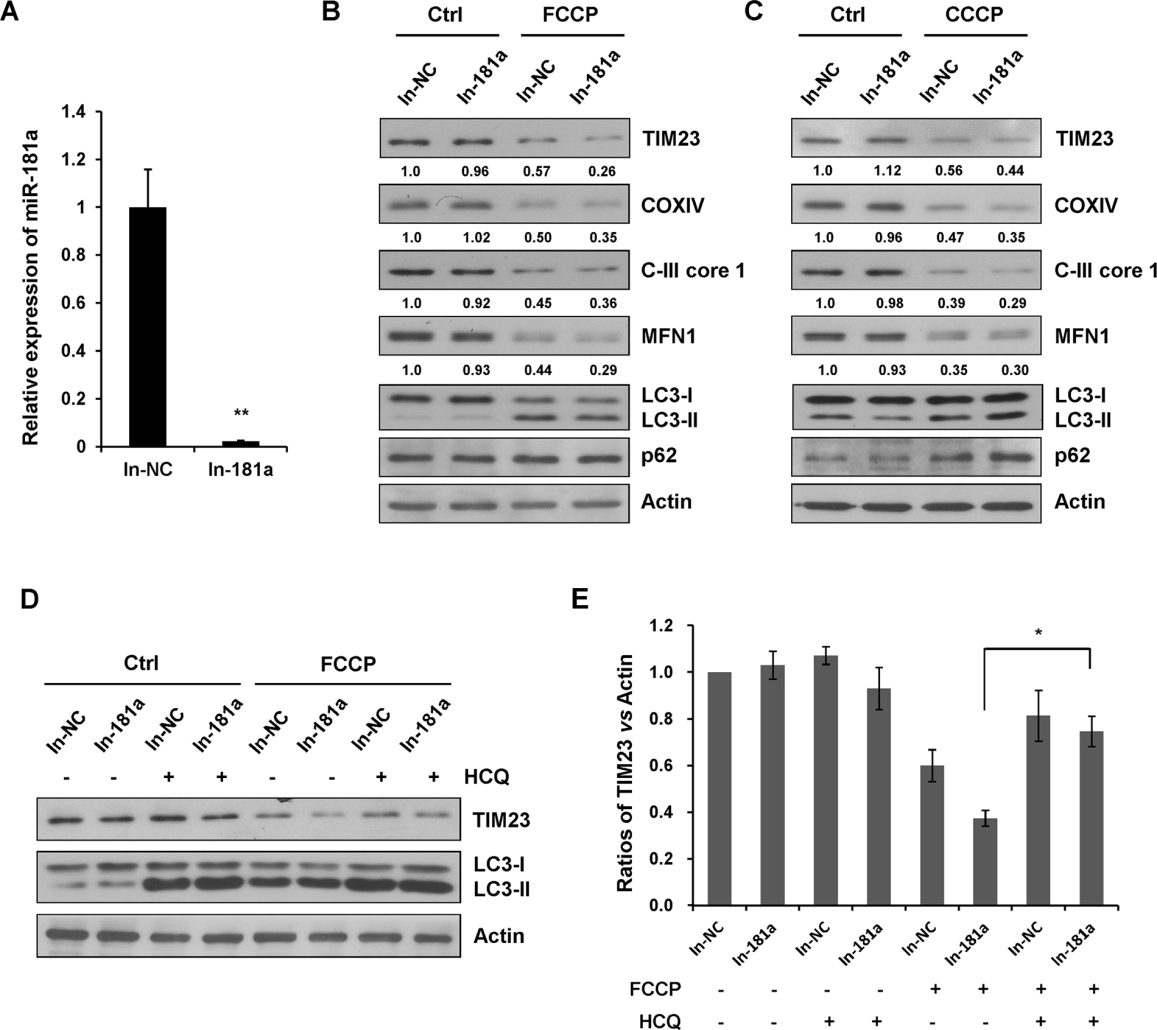
Inhibition of endogenous miR-181a promotes mitophagy **A.** The relative expression of miR-181a (compared with U6) in SH-SY5Y cells transfected with miR-181a inhibitor (In-181a) or the negative control (In-NC). **B.** SH-SY5Y cells transfected with In-181a or In-NC were treated with 10 μM FCCP or **C.** CCCP for 12 h. Samples were collected for western blot to analyze the expression of mitochondrial marker proteins, LC3, p62, and Actin. Densitometric ratios of TIM23/Actin are quantified by Image J software. **D.** SH-SY5Y cells transfected with In-NC or In-181a were exposed to FCCP (10 μM) and HCQ (20 μM) for 12 h. Western blot was performed to analyze the status of TIM23, LC3 and Actin. **E.** Image J densitometric analysis of the TIM23/Actin ratios from immunoblots is shown (data from three independent experiments, *p<0.05).

To test whether the loss of endogenous miR-181a would have a positive effect on mitophagy, a specific miR-181a antagomir (In-181a) was used to counteract the miRNA effects by specially paring mature endogenous miR-181a (Figure 4A). Upon FCCP or CCCP treatment, miR-181a inhibitor accelerated the degradation of TIM23, COXIV, C-III core 1, and MFN1 without affecting the LC3-I to LC3-II transition (Figure 4B & 4C). When cells were treated with HCQ, FCCP-induced degradation of TIM23 was markedly prevented in cells transfected with the miR-181a inhibitor, suggesting that the blockage of endogenous miR-181a promotes a strong autophagic flux (Figure 4D & 4E). We also performed immunoblot analysis in A172 cells to determine the effect of miR-181a inhibitor on mitophagy. Similarly, blockage of endogenous miR-181a accelerated FCCP-induced mitophagy in A172 cells (Supplementary Figure S2B).

### MiR-181a directly targets parkin E3 ubiquitin ligase

**Figure 5:**
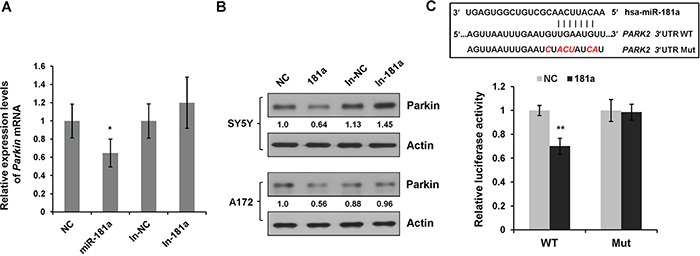
miR-181a directly targets E3 ubiquitin ligase Parkin **A.** Relative *Parkin* mRNA levels (compared with *Actin*) in SH-SY5Y cells transfected with miR-181a mimics or inhibitors were analyzed by quantitative RT-PCR. Data are represented as means±s.d. from three independent experiments, and analyzed using the student *t* test (**p<0.01). **B.** SH-SY5Y and A172 cells were transfected with NC, miR-181a, In-NC, In-181a, respectively. At 48 h after transfection, cell lysates were subjected to western blot analysis for Parkin and Actin. **C.** Diagram of the predicted miR-181a binding sites in *Parkin* 3′UTR. Luciferase reporter constructs containing WT or Mut *Parkin* 3′UTR were co-transfected with NC or miR-181a. Relative luciferase activity was normalized to NC. Data shown are means±s.d. from three independent experiments, **p<0.01.

To clarify the molecular mechanism of miR-181a on mitophagy, we searched for genes that contain potential miR-181a binding sites in their 3′UTRs using the miRNA targets prediction algorithms TargetScan, miRanda, and FindTar. Interestingly, *PARK2* (Parkin RBR E3 ubiquitin protein ligase) was identified as a putative miR-181a target. To confirm this prediction, we performed quantitative RT-PCR to examine the mRNA levels of Parkin. Overexpression of miR-181a significantly reduced the transcript levels of Parkin, whereas miR-181a increased Parkin mRNA expression (Figure 5A). Immunoblotting analysis revealed that overexpression of miR-181a resulted in a potent downregulation of endogenous Parkin protein expression in both SH-SY5Y and A172 cells. In contrast, introduction of antigomir miR-181a (In-181a) caused a modest increase in Parkin protein expression when compared with the control (In-NC) cells (Figure 5B).

To validate, we also checked the mRNA and protein expression levels of Parkin in SH-SY5Y cells that stably expressing miR-181a by lentiviral infection (Supplementary Figure S3A). Similar to transient transfection, stable expression of miR-181a markedly suppressed Parkin mRNA and protein expression, as well as FCCP-induced TIM23 protein degradation (Supplementary Figure S3B & S3C).

Next, we performed a luciferase activity assay to determine whether miR-181a directly regulated Parkin gene expression. We cloned the 3′UTR fragment containing the predicted miR-181a binding sites from human *PARK2* gene into a dual luciferase reporter system. As shown in Figure 5C, miR-181a markedly reduced luciferase activity for *PARK2* wild type luciferase reporter construct. However, disruption of the miR-181a binding sites prevented the susceptibility of the mutated luciferase reporter to miR-181a.

### Overexpression of miR-181a or silencing parkin prevents mitochondrial degradation

**Figure 6:**
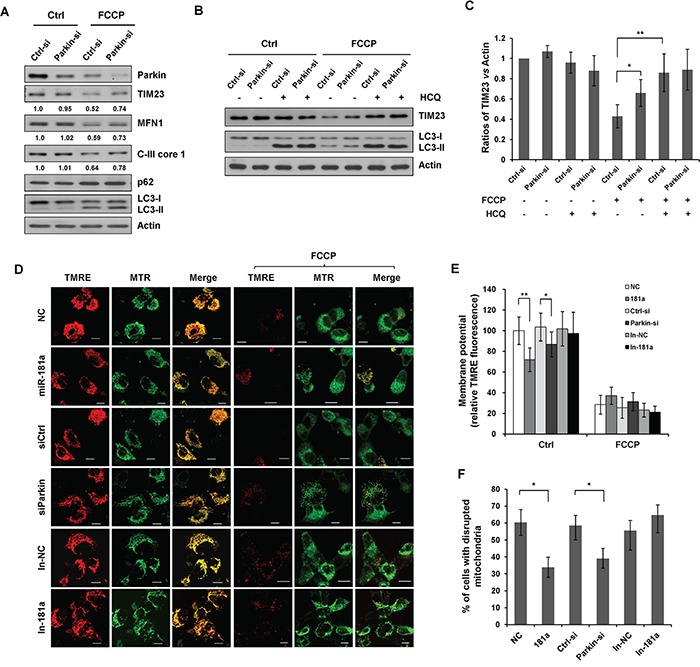
Silencing endogenous Parkin inhibits mitophagy **A.** SH-SY5Y cells were transfected with control or Parkin siRNA for 36 h. Cells were treated with 10 μM FCCP for 12 h. Sample were collected for western blot to analyze the expression of TIM23, MFN1, Parkin, C-III core 1, p62, LC3 and Actin. **B.** SH-SY5Y cells transfected with control or Parkin siRNA were exposed to FCCP and HCQ for 12 h. Western blot was performed to analyze the status of TIM23, LC3 and Actin. **C.** Image J densitometric analysis of the TIM23/Actin ratios from immunoblots is shown (data from three independent experiments, *p<0.05, **p<0.01). **D.** SH-SY5Y cells transfected with miR-181a, Parkin siRNA, In-181a, or the controls were treated with 10 μM FCCP for 12 h. Representative confocal images of TMRE and MitoTracker Green FM (MTR) are shown. Scale bar, 10 μm. **E.** Quantification of TMRE signals in cells before and after FCCP treatment. Data shown are means±s.d. from three independent experiments, *p<0.05, **p<0.01. **F.** The morphology of mitochondria was visualized by MitoTracker Green FM. The number of cells show disrupted mitochondria was quantified. All data are expressed as means±s.d., *p<0.05.

To examine the effect of miR-181a targets on mitophagy, we knocked down endogenous Parkin using Parkin-specific siRNA. Parkin siRNA impaired the FCCP-induced degradation of TIM23, C-III core 1, and MFN1 (Figure 6A). HCQ markedly prevented the FCCP-induced degradation of TIM23 in control (NC) cells without significantly affecting TIM23 levels in cells transfected with Parkin siRNA, indicating that Parkin siRNA suppressed FCCP-induced mittophagy (Figure 6B & 6C). We next investigated the effects of miR-181a and Parkin siRNA on mitochondrial membrane potential (ΔΨm) and mitochondrial morphology. Mitochondrial membrane potential was quantified by TMRE, a cationic fluorescent dye that accumulates inside the mitochondrial matrix according to the membrane potential present. FCCP treatment resulted in a marked reduction of membrane potential in all control cells and cells transfected with miR-181a, Parkin siRNA or miR-181a antagomir (In-181a). Interestingly, both miR-181a and Parkin siRNA caused a significant decrease of membrane potential in untreated cells (Figure 6D & 6E). Mitochondrial morphology was determined by MitoTracker Green FM, a fluorescent mitochondrial stain that is independent of the mitochondrial membrane potential. Treatment of FCCP resulted in a dramatic decrease in the number of mitochondria and disruption of mitochondrial network. We quantified cells with disrupted mitochondria, a significant difference in the percentage of cells with disrupted mitochondria was observed between control cells and cells transfected with miR-181a or Parkin siRNA. However, miR-181 antagomir, had a similar effect to the control cells in response to FCCP treatment (Figure 6D & 6F).

### Re-expression of parkin restores mitophagy inhibited by miR-181a

**Figure 7:**
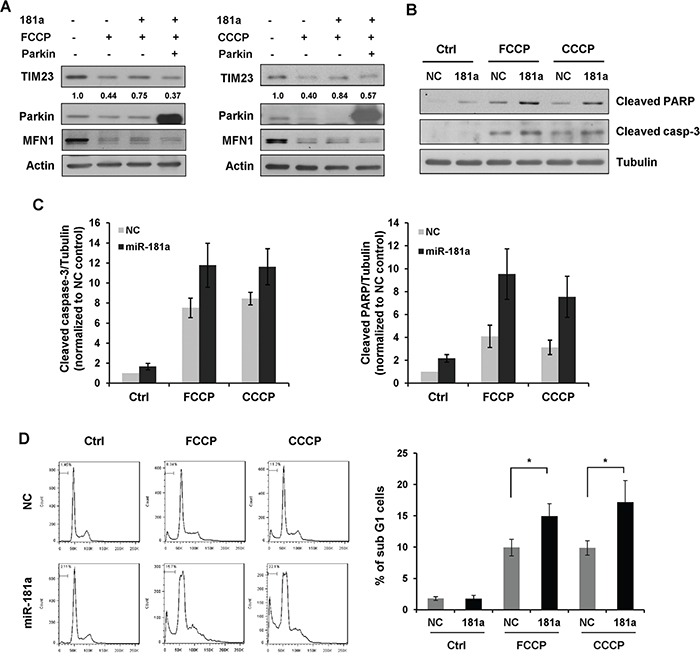
miR-181a sensitizes neuroblastoma cells to apoptosis induced by mitochondrial uncouplers **A.** Overexpression of Parkin restores the inhibitory effect of miR-181a on mitophagy. SH-SY5Y cells were transfected with miR-181a and Parkin plasmid as indicated. Cells were then cultured in the presence or absence of FCCP (10 μM) or CCCP (10 μM) for 12 h. Samples were collected for western blot to analyze the protein expression of TIM23, Parkin, MFN1 and Actin. **B.** SH-SY5Y cells transfected with miR-181a or NC were treated with 25 μM FCCP or CCCP for 24 h. Western blot was performed to analyze the status of cleaved caspase-3, cleaved PARP and Tubulin. **C.** Densitomeric analysis of cleaved caspase-3/Tubulin and cleaved PARP/Tubulin protein ratios. **D.** Flow cytometry analysis of sub G1 population in SH-SY5Y cells treated with FCCP or CCCP for 24 h. Data shown are means±s.d. from three independent experiments, *p<0.05.

To confirm the repression of Parkin was caused by the mitophagy-related effects of miR-181a, we performed a rescue experiment. miR-181a suppressed the loss of mitochondrial protein TIM23 upon treatment of FCCP or CCCP, and the re-introduction of exogenous Parkin lacking the miR-181a recognition elements induced greater mitophagy even in the presence of miR-181a. Therefore, the inhibitory effect of miR-181a on mitophagy was reversed by the re-expression of Parkin (Figure 7A).

### MiR-181a sensitizes neuroblastoma cells to apoptosis induced by mitochondrial uncouplers

Accumulating evidence has demonstrated that the PINK1/Parkin pathway is critical for the autophagic degradation of damaged mitochondria. Therefore, we were keen to know whether miR-181a mediated suppression of mitophagy might contribute to cell death following treatment with mitochondrial uncouplers. To test this, SH-SY5Y cells were transfected with miR-181a or NC, followed by treatment with 25 μM FCCP or CCCP for 24 h. Immunoblotting analysis revealed that both FCCP and CCCP caused an obvious increase in cleaved caspase-3 and PARP levels, indicating that mitochondrial uncouplers induces apoptotic cell death. More cleaved caspase-3 and PARP were detected in miR-181a transfected cells compared with NC controls (Figure 7B & 7C). To confirm these results, we examined sub G1 phase cells by flow cytometry. A small fraction of the sub G1 population was observed in control cells treated with FCCP or CCCP. Overexpression of miR-181a increased the sensitivity of SH-SY5Y cells to mitochondrial uncouplers (Figure 7D).

We next examined whether silencing Parkin might enhance mitochondrial uncoupler-induced cell death. Similar to that observed with miR-181a, Parkin siRNA increased caspase-3 cleavage compared with controls (Supplementary Figure S4A). Finally, we determined whether knocking down endogenous miR-181a might reduce the sensitivity of SH-SY5Y cells to mitochondrial uncouplers. In agreement with this prediction, miR-181a inhibitors markedly reduced the level of cleaved caspase-3 compared with control cells (Supplementary Figure S4B). Taken together, these results indicate that Parkin-mediated mitophagy is a survival mechanism as inhibition of Parkin-mediated mitophagy sensitized neuroblastoma cells to mitochondrial uncoupler-induced apoptosis.

## DISCUSSION

Recently, several miRNAs have been well characterized with regards to the modulation of different autophagic processes. However, little is known about the role of miRNAs in regulating mitophagy. In this study, we used human neuroblastoma SH-SY5Y cells as an *in vitro* model to determine the role of miR-181a in mitophagy and apoptosis. Interestingly, we show that miR-181a is responsive to the mitochondrial uncoupling agents FCCP and CCCP in SH-SY5Y and A172 cell lines as well as primary human neurons. Overexpression of miR-181a suppresses mitochondrial uncoupler-induced mitophagy by inhibiting the degradation of mitochondrial proteins TIM23, COXIV, complex III core subunit, and MFN1 without affecting global autophagy. Knocking down of endogenous miR-181a accelerates the autophagic degradation of damaged mitochondria. miR-181a directly targets Parkin, an E3 ubiquitin ligase that ubiquitinates a series of mitochondrial substrates and contributes to mitochondrial clearance through autophagy. Overexpression of exogenous Parkin restored the inhibitory effect of miR-181a on mitophagy. We further demonstrate that miR-181a sensitizes SH-SY5Y neuroblastoma cells to apoptosis induced by mitochondria uncouplers. When taken together, the findings we present in this study establish an important link between microRNA and Parkin-mediated mitophagy.

It has been reported that miR-181a is enriched in brain and their aberrant expression has been associated with brain diseases [39–41]. miR-181a is overexpressed and acts as a marker of bad prognosis for aggressive neuroblastoma and to aid the comparison of neuroblastoma with low-grade stages [42]. Mitophagy has a crucial function in controlling mitochondria quality, defects in mitophagy have been implicated in a variety of human disorders including cancer; therefore we propose that miR-181a mediated repression of mitophagy may contribute to the loss of cellular homeostasis and tumor development.

Mitochondrial membrane potential (ΔΨm) is critical for maintaining the physiological function of the respiratory chain to generate ATP. Depolarized mitochondria are unable to reestablish membrane potential and excluded and targeted for mitophagy. Recent studies demonstrated that PINK1 and Parkin are involved in the maintenance of mitochondrial morphology and membrane potential. Reduced transmembrane potential in *PINK1* deficient cells has been reported in a wide variety of cells [43–46]. Parkin also plays a role in mitochondrial dynamics, presumably related to its E3 ubiquitin ligase activity. The mitochondrial membrane potential was significantly lower in siRNA-mediated *Parkin* knockdown cells [47]. In line with this effect, we also observed that both Parkin siRNA and miR-181a decreased the mitochondrial membrane potential in SH-SY5Y cells. The mitochondrial membrane potential decrease in miR-181a transfected cells might be mediated by downregulation of Parkin and the decreased ubiquitin ligase activity.

Increasing evidence has indicated that Parkin shows protective function against apoptosis [48–50]. In agreement with previous reports, our study demonstrates that both miR-181a and Parkin siRNA sensitize SH-SY5Y cells to apoptotic cell death in response to mitochondrial uncoupling agents, whereas miR-181a inhibitor prevents cell death. On the other hand, it has been reported that miR-181a could target the anti-apoptotic Bcl-2 family members Mcl-1 and Bcl-2, overexpression of miR-181a increases glucose deprivation-induced apoptosis in astrocytes [51]. miR-181a also sensitizes non-small cell lung cancer A549 cells to the lethal action of cisplatin by stimulating Bax oligomerization and the activation of proapoptotic caspases [52]. In this regard, it is particularly noteworthy that miR-181a mediated downregualtion of Parkin as well as other potential targets may contribute to mitochondrial uncoupler-induced apoptosis. Our findings thus provide a potential therapeutic strategy for neuroblastoma and neurodegenerative diseases.

## MATERIALS AND METHODS

### Cell culture and treatment

The human neuroblastoma cell line SH-SY5Y, the human glioblastoma cell lines A172 and U251 were obtained from the American Type Culture Collection. Both cell lines were grown in Dulbecco's modified Eagle's medium (Invitrogen), supplemented with 10% fetal bovine serum (BioWest, S1820-500), 10 U/ml penicillin/streptomycin (Invitrogen). Human neurons (HN) were purchased from ScienCell Research Laboratories, cells were grown in Neuronal medium supplemented with neuronal growth supplement (ScienCell, 1562) and penicillin/streptomycin (ScienCell, 0503). All the cells were cultured in a 5% CO2-humidified incubator at 37°C. CCCP (Sigma, C2759), FCCP (Sigma, C2920), and hydroxychloroquine (HCQ) (Sigma, H0915) were dissolved in dimethyl sulfoxide (DMSO). To induce mitophagy, cells were treated with FCCP or CCCP for the designated time points.

### MicroRNAs, siRNAs, plasmids and transfection

MiR-181a, NC, miR-181a inhibitor (In-181a) and control miRNA inhibitor (In-NC) were synthesized by GenePharma (Suzhou, China). Parkin siRNA was purchased from QIAGEN (CA, USA). Parkin plasmid was purchased from Addgene (#17613). miRNAs, miRNA inhibitors, siRNA or plasmid were transfected into SH-SY5Y cells using Lipofectamine™ 2000 (Invitrogen, 11668-019) according to the manufacturer's protocol. The sequences of siRNAs are as follows: *PARK2* (5′-GUUUGUUCACGACCCUCAATT-3′); Scramble control (5′-UUCUCCGAACGUGUCACGU-3′).

To obtain SH-SY5Y stable cell line, we purchased lentiviruses from GeneChem (Shanghai, China), in which the plasmids (GV235) contain the fragment of pre-*MIR181A*. A lentiviral vector expressing a scrambled RNA was used as negative control. SH-SY5Y cell were infected with viruses along with 5ug/ml polybrene and selected stable cell lines with 10ug/mL puromycin (Gibco, A11138-03).

### qRT-PCR gene expression analysis

Total RNA was isolated with RNA iso Plus (Takara, D9108B) according to the manufacturer's instructions. Relative gene expression was determined using quantitative RT-PCR. Total RNA was reverse-transcribed using reverse transcription kit (Takara, D6130). Quantitative PCRs were performed using SYBR Green PCR Master Mix (Toyobo, QPK-201). Relative mRNA levels were normalized to ACTB. Primers used were: 5′-GGAAATCGTGCGTGACATTAA-3′ (ACTB, forward); 5′-TCGAGGCAGCTCGTAGCTCTT-3′ (ACTB, reverse); 5′-CCCACCTCTGACAAGGAAACA-3′ (PARK2, forward); 5′-TCGTGAACAAACTGCCGATCA-3′ (PARK2, reverse). qRT-PCRs of miRNAs were performed according to the manufacturer's instruction using a TaqMan MicroRNA Reverse Transcription Kit (Applied Biosystems, 43366597) and Real-time PCR Master Mix (Toyobo, QPK-101). MiRNA probes were: has-miR-181a (Applied Biosystems, 000480), RNU6 (Applied Biosystems, 001093). Fold-changes were calculated using the ΔΔ*Ct* method with normalization to RNU6.

### Measurement of mtDNA content

Genomic DNA used for the analysis of mtDNA content was isolated from SH-SY5Y cells using TaKaRa MiniBEST Universal Genomic DNA Extraction kit (TaKaRa Bio Inc.). The relative mtDNA copy number was determined by qPCR with primers for the mitochondrial *16S rRNA* gene and the nuclear *Actin* gene. PCR amplification was performed with the qTOWER 2.0 Real Time PCR Thermal Cycler (Analytik Jena) in 20 μL volume containing 50 nM of each primer, 10 μL SYBR Green PCR Master Mix for detection and 20 ng DNA as template. All PCR reactions were performed in triplicates. Primers used were: 5′-GGTGCAGCCGCTATTAAAGG-3′ (16S rRNA, forward); 5′-ATCATTTACGGGGGAAGGCG-3′ (16S rRNA, reverse); 5′-TGACGTGGACATCCGCAAAG-3′ (ACTB, forward); 5′-CTGGAAGGTGGACAGCGAGG-3′ (ACTB, reverse).

### Quantitative GFP-LC3 analyses

SH-SY5Y cells were transfected with miR-181a or control miRNA (NC) for 48 h. The transfected cells were treated with FCCP (10 μM) or CCCP (10 μM) for 6 h to induce mitophagy. The number of GFP-LC3 dots in each sample was counted from at least 150 cells from randomly placed positions within each sample.

### Western blotting

Cells were lysed in ice-cold whole cell extract buffer (50 mM TRIS-HCl, pH 8.0, 4 M urea and 1% Triton X-100), supplemented with complete protease inhibitor mixture (Roche Diagnostics, 04693132001). The whole cell extracts were resolved by SDS-PAGE gel electrophoresis, and transferred to nitrocellulose membranes. Membranes were probed with the indicated primary antibodies followed by appropriate HRP-conjugated secondary antibodies (KPL). Protein bands were visualized using ECL Blotting Detection Reagents (Thermo Scientific, 32106). Primary antibodies used for western blotting were as follows: Parkin (Cell Signaling, 4211), LC3B (Sigma, L7543), MFN1 (Abcam, ab57602), TIM23 (Proteintech, 11123-1-AP), COXIV (Cell Signaling, 4844), C-III core 1 (Invitrogen, 459140), p62 (MBL, PM045), cleaved Caspase-3 (Cell signaling Technology, 9664), cleaved PARP (Cell signaling Technology, 5625), Actin (Proteintech, 60008-1-IG), α-Tubulin (Sigma, T9026).

### Flow cytometry

The mitochondrial mass was determined by flow cytometric analysis using Nonyl Acridine Orange (NAO, Invitrogen, A-1372). SH-SY5Y cells were transfected with miR-181a or NC, then treated with 10 μM FCCP for 12 h. Cells were incubated for 30 min with 2.5 μM NAO and washed with PBS. Cells were then harvested and counted using a BD Influx™ (BD Biosciences) flow cytometer.

### Luciferase activity assay

The 3′ UTR of the human *PARK2* gene (WT) was PCR amplified from human cDNA using the following primers: 5′-AGCGAGCTC GCATCTATTCCCAAAGAACCCC-3′ (WT, forward); 5′-GCTCTAGAAGTGGAGCCAAATGTATTAGCAA-3′ (WT, reverse) and cloned into the pmirGLO dual-luciferase miRNA Target expression vector (Promega, 9PIE133). The corresponding mutant construct (Mut) was created by mutating the seed region of the miR-181a binding sites. The primers used for cloning mutant plasmid were: 5′-TCATTGAATTACAGGGAAGAAATC-3′ (Mut, forward); 5′-TAGTAGATTCAAATTAACTTTCAT-3′ (Mut, reverse).

The luciferase reporter vectors containing WT or Mut 3′UTR were cotransfected with miR-181a or NC control into SH-SY5Y cells using Lipofectamine™ 2000 (Invitrogen, 11668-019). Cells were lysed at 36 h after transfection, firefly luciferase and renilla luciferase activities were measured by a dual-luciferase reporter system (Promega, E1960). Data shown are representative of three independent experiments.

### Measurement of mitochondrial membrane potential

Mitochondrial membrane potential was measured using the fluorescent dye Tetramethylrhodamine ethyl ester (TMRE) (Invitrogen, T-669). SH-SY5Y cells transfected with miR-181a, Parkin siRNA, In-181a or the controls were treated with or without FCCP (10 μM) for 6 h, then incubated with TMRE (25 nM) and MitoTracker Green FM (200 nM) for 30 min at 37°C. The relative fluorescence intensity of TMRE was quantified by Image J software, data shown are representative of three independent experiments.

### Live-cell imaging and colocalization analysis

SH-SY5Y cells grown in a 35-mm glass bottom plate (MatTek, P35G-1.5-14-C) were co-transfected with GFP-LC3 and CellLight Mitochondria-RFP (Invitrogen, C10505). At 24 h after transfection, cell images of GFP-LC3 and Mito-RFP were captured with an Olympus FV1000 confocal microscope. To quantify the colocalization between GFP-LC3 and Mito-RFP, more than 100 cells expressing both Mito-RFP and GFP-LC3 were calculated according to the number of mitochondria-localized LC3 puncta.

To determine the colocalization between mitochondria and lysosomes, SH-SY5Y cells were loaded with 200 nM MitoTracker Green FM (Invitrogen, M7514) and 25 nM LysoTracker Red DND-99 (Invitrogen, L-7528) for 20 min at 37°C. Cell images were obtained with an Olympus FV1000 confocal microscope. The colocalization between mitochondria and lysosomes was calculated according to the number of mitochondria-localized lysosomes.

### Statistical analyses

Data are expressed as mean ± s.d. of three independent experiments. Statistical analyses were performed using two-tailed student's *t*-test. Differences were considered significant with a value of *p*<0.05.

## SUPPLEMENTARY FIGURES


