# Supplementary material for: MicroRNA-181a suppresses parkin-mediated mitophagy and sensitizes neuroblastoma cells to mitochondrial uncoupler-induced apoptosis
Source: Oncotarget. 2016 Jun 2;7(27):42274–87. doi: 10.18632/oncotarget.9786 (PMC5173134; doi:10.18632/oncotarget.9786)
Supplement: Supplementary file 1 [file oncotarget-07-42274-s001.pdf]

SUPPLEMENTARY FIGURES

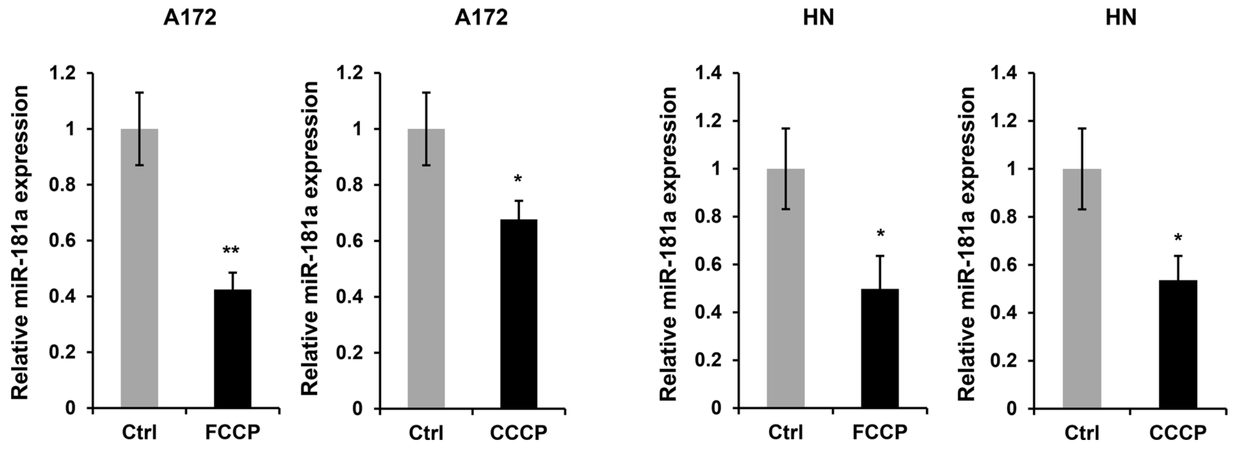

Supplementary Figure S1: miR-181a is downregulated by mitochondrial uncouplers. Analysis of miR-181a expression in human glioblastoma cell line A172 and primary human neurons (HN). Cells were treated with 10 µM FCCP or CCCP for 6 h. Data shown are from three independent experiments, **p*<0.05, ***p*<0.01.

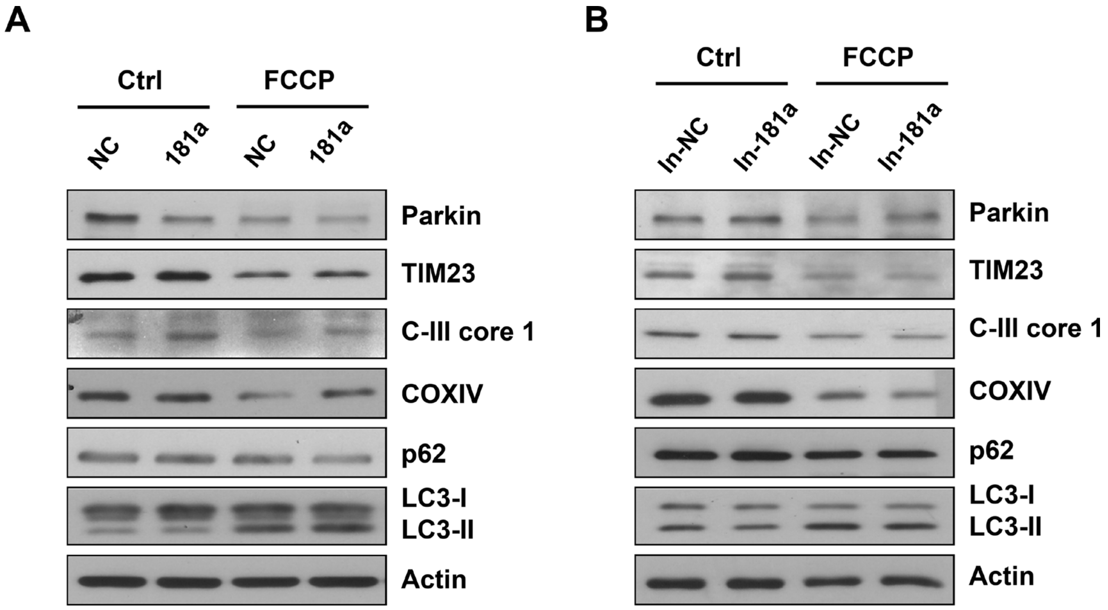

Supplementary Figure S2: The effect of miR-181a on mitochondrial uncoupler-induced mitophagy. A. A172 cells transfected with miR-181a or NC were treated with 10 µM FCCP for 12 h. Samples were collected for western blot to analyze the expression of TIM23, COXIV, C-III core, MFN1, LC3, p62, and Actin. **B.** A172 cells transfected with In-181a or In-NC were treated with 10 µM FCCP for 12 h. Samples were collected for western blot analysis.

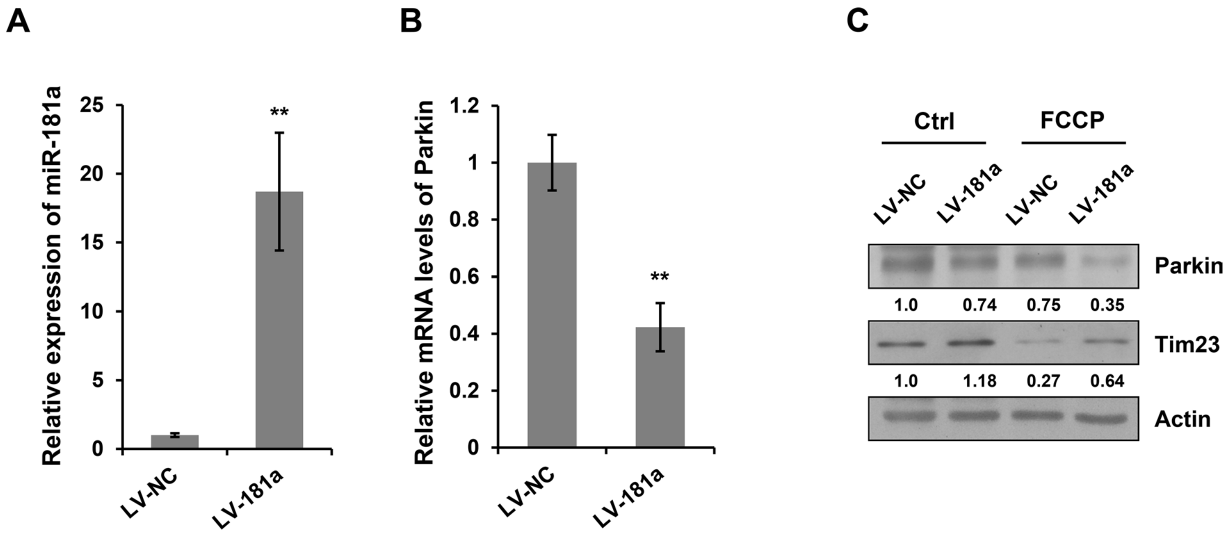

Supplementary Figure S3: Stable transfection of miR-181a inhibits parkin expression and mitophagy. A. Analysis of miR-181a expression in SH-SY5Y cells stably expressing miR-181a (Lv-181a) by lentiviral infection. **B.** Relative *Parkin* mRNA levels (compared with *Actin*) in SH-SY5Y cells stably expressing miR-181a (Lv-181a) or NC (Lv-NC) were analyzed by quantitative RT-PCR. Data are represented as means±s.d. from three independent experiments, and analyzed using the student *t* test (**$p<0.01$). **C.** SH-SY5Y cells stably expressing miR-181a or NC were treated with 10 µM FCCP for 12 h. Samples were collected for western blot to analyze the expression of TIM23, Parkin, and Actin. Image J densitometric analysis of the TIM23/Actin and Parkin/Actin ratios from immunoblots is shown.

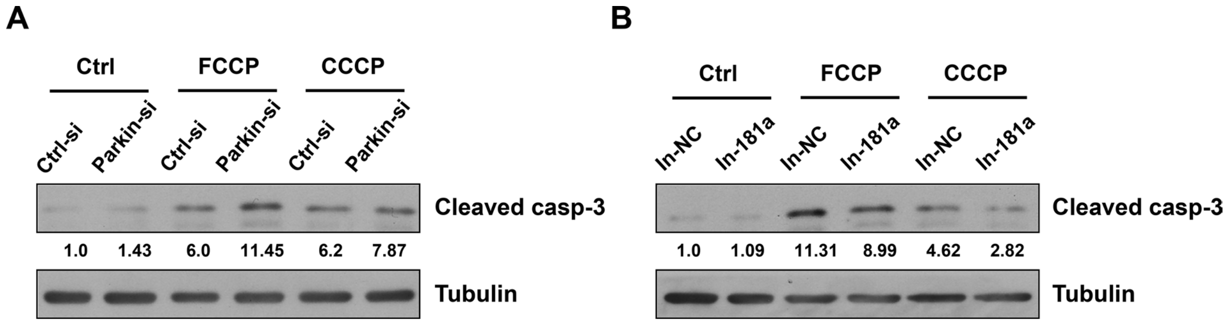

Supplementary Figure S4: The effects of Parkin siRNA and miR-181a antagomir on mitochondrial uncoupler-induced apoptosis. A. SH-SY5Y cells were transfected with control or Parkin siRNA, **B.** In-NC or In-181a and then treated with 25 µM FCCP or CCCP for 24 h. Samples were collected for western blot to analyze the status of cleaved caspase-3 and Tubulin.
